# Opening the Schrödinger Box: Short- and Long-Range Mammalian Heart Rate Variability

**DOI:** 10.3389/fphys.2021.665709

**Published:** 2021-06-30

**Authors:** Ido Weiser-Bitoun, Moran Davoodi, Aviv A. Rosenberg, Alexandra Alexandrovich, Yael Yaniv

**Affiliations:** ^1^Biomedical Engineering Faculty, Technion-IIT, Haifa, Israel; ^2^Computer Science Faculty Technion-IIT, Haifa, Israel

**Keywords:** animals, cardiac disease, sinoatrial node, autonomic blockade, ECG

## Abstract

**Background:**

The interactions between the autonomic nervous system (ANS), intrinsic systems (e.g., endocrine), and internal pacemaker mechanisms govern short (milliseconds–seconds)- and long (seconds–minutes)-range heart rate variability (HRV). However, there is a debate regarding the identity of the mechanism underlying HRV on each time scale. We aim to design a general method that accurately differentiates between the relative contribution of the ANS and pacemaker mechanisms to HRV in various mammals, without the need for drug perturbations or organ isolation. Additionally, we aim to explore the universality of the relative contribution of the ANS and pacemaker system of different mammals.

**Methods:**

This work explored short- and long-range HRVs using published ECG data from dogs, rabbits, and mice. To isolate the effects of ANS on HRV, ECG segments recorded before and after ANS-blockade were compared.

**Results:**

Differentiation of the ANS from extrinsic and intrinsic pacemaker mechanisms was successfully achieved. In dogs, the internal pacemaker mechanisms were the main contributors to long-range and the ANS to short-range HRV. In rabbits and mice, the ANS and the internal pacemaker mechanisms affected both time scales, and anesthesia changed the relative contribution of the pacemaker mechanism to short- and long-range HRVs. In mice, the extrinsic mechanisms affected long-range HRV, while their effect was negligible in rabbits.

**Conclusion:**

We offer a novel approach to determine the relative contributions of ANS and extrinsic and intrinsic pacemaker mechanisms to HRV and highlight the importance of selecting mammalian research models with HRV mechanisms representative of the target species of interest.

## Introduction

Beat-to-beat variability in heart rate is primarily the result of the integration of intrinsic stochastic heart pacemaker (i.e., the sinoatrial node) mechanisms, extrinsic systems, such as hormones and external autonomic nervous system (ANS) activity. This integration involves a mix of (i) stochastic release of neurotransmitters that regulate channels on the cell membrane (Yang and Xu-Friedman, 2013), and (ii) stochastic opening and closing of membrane channels and sarcoplasmic reticulum receptors (Adair, 2003). Note that a change in channel kinetics can be affected by neurotransmitter and hormone concentrations and that channel and receptor kinetics can be affected by internal signaling (i.e., Ca^2+^ and post-translation modification signaling). As there are three stochastic main systems and also mutual entrainment between the ANS, heart pacemaker, and pacemaker cell synchronization, heart rate variability (HRV) can be observed in short (milliseconds–seconds) and long (seconds–minutes) time scales which differ between animals.

Because heart and respiratory diseases can be characterized by changes in short- and long-range HRVs, it is important to characterize the specific system failures that underlie these changes. Studies have used pharmacological ANS blockers to isolate the role of ANS from the extrinsic and intrinsic pacemaker systems (Akselrod et al., 1981; Rubini et al., 1993; Frey et al., 1996; Zwiener et al., 1996; Just et al., 2000). We recently designed a filter that removes the ANS contribution from basal dog ECG data without the need for an ANS blocker or organ isolation (i.e., “opening the Schrödinger box”) (Rosenberg et al., 2020). The filter enables isolation of the individual contributions of the ANS and extrinsic and intrinsic pacemaker systems to HRV. However, it remains to be determined if a general filter can be applied to other mammals, where the ANS contribution to heart rate may be different. This study aimed to (i) test whether a general filter that isolates the contribution of the ANS from those of the intrinsic and internal pacemaker mechanisms can be applied to mammals, specifically to dogs, mice, and rabbits, which are common experimental models and to (ii) explore the universality of the relative contribution of the ANS and extrinsic and intrinsic pacemaker systems (that have different degree of ANS activation and average heart rate) to short- and long term HRV across mammals. Resolving the first aim will enable the use of this noninvasive tool for the identification of specific system failures and appropriate treatments. Resolving the second aim will allow us to understand the generalizability of conclusions drawn from animal models with respect to human physiology and reduce the need for animal models in preclinical and basic science research studying these biological phenomena.

## Materials and Methods

### Data Sources

Published ECG data collected from dogs (Billman et al., 2015), rabbits (Yaniv et al., 2014), mice (Yaniv et al., 2016; Moen et al., 2019), and humans [Normal Sinus Rhythm Database (Goldberger et al., 2000)] were used. The protocols and experimental procedures were approved by the research committees overseeing the original studies described in the respective publications. The data were collected during the daytime. Both mice and rabbits were anesthetized with isoflurane and were breathing spontaneously throughout the experiment. Each recording included a basal segment (e.g., no drug intervention), followed by a segment collected under the influence of atropine and propranolol (for dog and mouse data) or after vagus nerve cut and hexamethonium administration (for anesthetized rabbit data). From each heartbeat interval segment with a duration of 10 min, up to 5, 4, or 3 min for humans, dogs, rabbits, and mice, respectively, were used. For dog, anesthetized rabbit, awake mouse, and anesthetized mouse data, a 10-s sliding window low-pass filter at the beginning of the signal was compared to a threshold defined as the mean value of the data starting from 0.5, 1, and 2 min after the ANS blockade, for dogs, rabbits, and mice, respectively (this part of the segment was considered transient-free). When the sliding window average was within 5% of this threshold, the transient period was considered to be complete and the remainder of the segment was used. Because the heartbeat interval for awake (Ishii et al., 1996) or anesthetized mice (Lakin et al., 2018) was found to increase after autonomic blockade (ABK), only recordings that met this criterion were used.

### Heartbeat Interval Processing

When analyzing the heartbeat interval data, all three interval filtering methods provided by the PhysioZoo platform (Behar et al., 2018a, b) were used. The following configurations were applied: (a) For range-based filtering, intervals outside a range defined for each mammal were excluded (0.28–2.4, 0.25–1.2, 0.14–0.58, and 0.05–0.24 s for humans, dogs, rabbits, and mice, respectively), corresponding to specific heart rate ranges (25–214, 50–240, 103–429, and 250–1,200 bpm, respectively). (b) For the moving-average filter, a window size of 21 samples (10 samples on each side of the central sample) was used and the sample was taken out if its value exceeded 20% of the average of the window. (c) For quotient filtering, a value of *r* = 0.8, corresponding to an 80% tolerance, was used when deciding to exclude an interval based on its predecessor or successor.

### Heart Rate Variability Metric Calculation

Processed heartbeat intervals were analyzed using the PhysioZoo platform (Behar et al., 2018b). The platform was used to calculate linear [SD of NN interval (SDNN)], frequency [very low frequency (VLF), low frequency (LF), and high frequency (HF)], and nonlinear (entropy) HRV metrics. As shown by Behar et al. (2018b) for mathematical definition and reference to the source.

### Internal Pacemaker Mechanism Signature

To determine the relative contribution of the ANS to HRV, we sought to apply a transformation to basal data to remove most of the ANS component, such that the resulting signal resembles (in terms of its entropy) ABK data (Supplementary Figure 1). For dog data, the basal information was in both short- and long-range HRVs, while the ABK information was mainly in the short-range time scale (as shown in the “Results” section). The HF band peak of the basal signal was around 0.37 Hz, which corresponds to the respiratory rate, and a lower peak is in the LF band signal at around 0.16 Hz. To apply a larger attenuation in a specific band, we used two concatenated finite impulse response (FIR) filters. The first one was a low-pass filter with a cutoff frequency of 0.1 Hz, while the second one was a band-stop filter applied in the range of 0.1–0.3 Hz. “Blackman” tapering (Thomson et al., 1976) of the order of 10 and 100 was used for both filters, respectively. To amplify the attenuation and to eliminate the phase (i.e., to receive only real values), zero-phase filtering with a sampling frequency of 1.754 Hz (adequate to the Shannon-Nyquist theorem) was applied. For anesthetized rabbit data, the majority of the information was in the short-range HRV (as shown in the “Results” section). The HF band peak of the basal signal at around 0.6 Hz, which corresponds to the respiratory rate, and another peak at around 1.0 Hz, were both diminished after ABK. An FIR band-stop filter with two bands at 0.08–0.3 and 0.4–1.1 Hz was used. “Blackman” tapering of the order of 200 was used. Again, to amplify the attenuation and eliminate the phase, we used zero-phase filtering with a sampling frequency of 2.3 Hz. For awake mice, the majority of the information was in long-range HRV, and its relative content increased after ABK (as shown in the “Results” section). We noted a peak in the LF band signal at around 0.5 Hz and a lower peak in the HF band signal at around 2.7 Hz, which corresponds to the respiratory rate. The LF peak was diminished and the HF band power decreased after ABK (as shown in the “Results” section). An FIR band-stop filter with two bands at 0.4–0.9 and 1.2–2.7 Hz, was used. “Blackman” tapering of the order of 250 and zero-phase filtering with a sampling frequency of 10 Hz were applied. For anesthetized mouse data, we noticed a substantial contribution of HF band power to the basal signal, which contributed mostly to the signal at ABK state, thus the power of the band was not filtered out. An FIR band-stop filter with one band on part of the LF band at 0.2–0.5 Hz was used. “Blackman” tapering of the order of 100 was used. Again, to amplify the attenuation and to eliminate the phase, zero-phase filtering with a sampling frequency of 10 Hz (double the mammal-specific resampling frequency of the RR intervals) was applied.

## Results

### Filtering the ANS Signature From Heartbeat Intervals of Awake Animals

We first compared mammals under awake conditions. To explore the role of internal pacemaker mechanisms and the external ANS on short- and long-range HRVs, heartbeat intervals derived from ECG recordings of awake dogs (*n* = 14, aged 1–4 years) (Billman et al., 2015) and awake mice (*n* = 5, aged 2–4 months) (Moen et al., 2019) collected under basal and ABK conditions, were analyzed.

**FIGURE 1:**
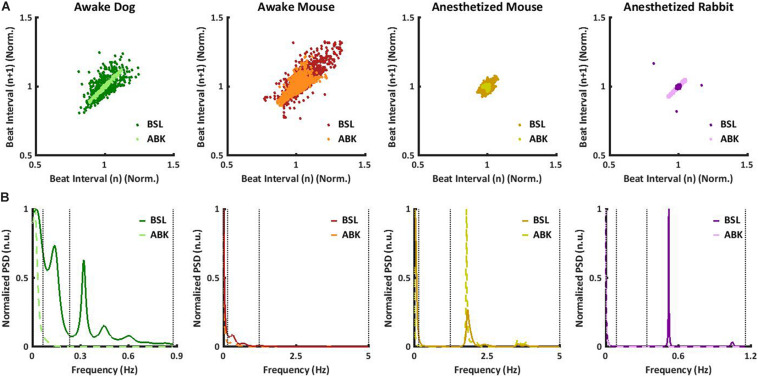
Mammalian heartbeat intervals in the basal (BSL) and autonomic nervous blockade (ABK) states. Representative examples, for each considered animal model, of **(A)** normalized Poincaré plot and **(B)** normalized power spectral density (PSD) before and after ABK. All the results of the graphs are from the same window (BSL and ABK).

**FIGURE 2:**
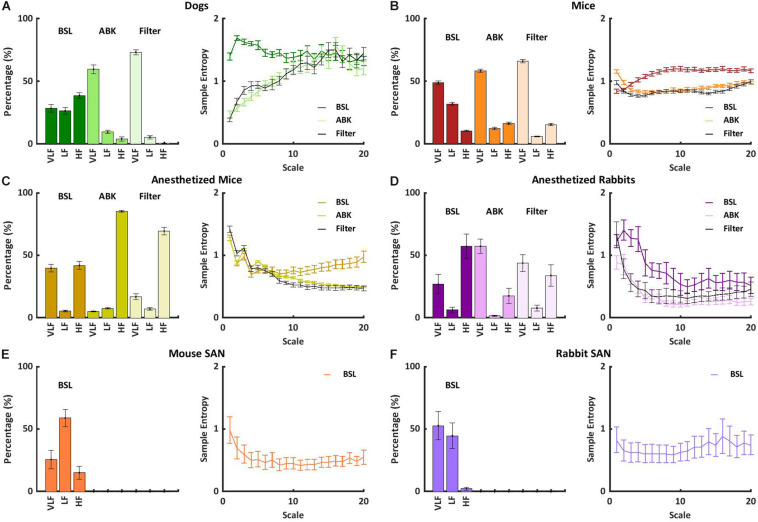
Short- and long-range heart rate variability (HRV) under BSL and ABK states. Normalized power in the very low frequency (VLF), low frequency (LF), and high frequency (HF) bands and average multiscale entropy in **(A)** dogs (*n* = 14, aged 1–4 years), **(B)** awake mice (*n* = 5, aged 2–4 months), **(C)** anesthetized mice (*n* = 6, aged 2–4 months), **(D)** anesthetized rabbits (*n* = 8, aged 2–4 months), **(E)** mouse *ex vivo* sinoatrial nodes (SANs) (*n* = 12, aged 2–4 months), and **(F)** rabbit *ex vivo* SANs (*n* = 9, aged 1–4 years). Frequency and multiscale entropy dog results of BSL and ABK are from Rosenberg et al. (2020).

Under basal conditions, the average heartbeat interval and SDNN of dogs were higher than that of mice (Table 1). Poincaré plots show the relationship between the current and the consecutive beat interval. Higher HRV is shown by the high scattering of the data. Figure 1A visualizes and Table 1 shows that the variance in the heartbeat interval decreased after ABK in dogs as compared to their basal conditions, while, in mice, it was not significantly changed in comparison to their respective basal states. Knowledge of the relative contributions of ANS and extrinsic and intrinsic pacemaker mechanisms to HRV on different time scales will enable the design of a filter that removes the ANS contribution from basal data. To this end, we explored the power spectral density (PSD) in different frequency bands. The VLF band represents the long-range time scale, while the LF and HF bands represent the short-range time scale. Note that the frequency range of VLF, LF, and HF is defined per-mammal (Behar et al., 2018a). ABK abolished the peaks in LF and HF bands of both dogs and mice (Figure 1B). Figures 2A,B show the relative PSD in different bands under both basal and ABK conditions in dogs and mice, respectively. In dogs, under the basal state, the power was distributed almost equally between all three bands. ABK led to a relative decrease in the HF and LF bands, leaving the majority of the normalized power in the VLF band. Thus, in dogs, the ANS contributes mainly to the short-range time scale, while the extrinsic and intrinsic pacemaker mechanisms contribute to the long-range time scale. In mice, half of the power under basal conditions resided in the VLF band, while the remainder was distributed between the LF and HF bands, at a ratio of 3:1. ANS blockade relatively reduced the power in the mouse LF band but did not abolish it as in dogs. It also increased the normalized PSD in the VLF but not in the HF bands. Thus, in mice, both short- and long-range time scales are affected by ANS and extrinsic and intrinsic pacemaker mechanisms.

**TABLE 1:** Heart rate variability (HRV) measures for different mammals.

	Dogs (*n* = 14)			Awake mice (*n* = 5)		
	BSL	ABK	Filter	BSL	ABK	Filter
AVNN (ms)	516.1 ± 17.9	460.3 ± 11.3	486.6 ± 13.4	110.3 ± 1.3	118.6 ± 1	110.5 ± 1.4
SDNN (ms)	73.4 ± 6.3	21.6 ± 2.1	31.5 ± 2.7	10.6 ± 0.3	4.7 ± 0.2	9.3 ± 0.4
Total power (ms^2^)	5,496 ± 988.9	247.4 ± 47.3	880.5 ± 233.4	108 ± 7.5	23.1 ± 2.6	79.7 ± 7.1
HF (ms^2^)	2,466.3 ± 544.9	12.5 ± 6.1	9.6 ± 3.3	10.8 ± 0.8	2.3 ± 0.2	10.3 ± 0.8
LF (ms^2^)	1,322 ± 223.6	18.8 ± 3.9	12.5 ± 1.9	29 ± 1.9	3.6 ± 0.5	3.6 ± 0.3
VLF (ms^2^)	1,405.7 ± 286.5	139.3 ± 30	779.9 ± 215.4	58.2 ± 5.6	14.4 ± 1.8	55.3 ± 5.6
HF norm ()	38.8 ± 2.4	4.3 ± 1.6	2.4 ± 0.9	10.7 ± 0.5	16.6 ± 1	15.7 ± 0.9
LF norm ()	26.7 ± 2.5	9.8 ± 1.3	2.7 ± 0.5	32.2 ± 1.2	12.6 ± 0.9	6.2 ± 0.4
VLF norm ()	28.7 ± 2.9	59.8 ± 3.4	85.6 ± 1.3	49 ± 1.4	58.6 ± 1.4	66.2 ± 1.3
HF norm by basal ()		0.4 ± 0.2	0.4 ± 0.2		2.1 ± 0.2	9.3 ± 0.7
LF norm by basal ()		0.5 ± 0.1	0.5 ± 0.1		3.7 ± 0.6	3.5 ± 0.3
VLF norm by basal ()		4.3 ± 1.3	29.1 ± 4.9		12.6 ± 1.3	49.9 ± 4.5

	**Anesthetized mice (*n* = 8)**			**Anesthetized rabbits (*n* = 7)**		
	**BSL**	**ABK**	**Filter**	**BSL**	**ABK**	**Filter**

AVNN (ms)	125.5 ± 0.8	131.3 ± 0.4	125.5 ± 0.8	192.8 ± 5.8	204.6 ± 3.7	192.8 ± 5.8
SDNN (ms)	2.5 ± 0.2	1.7 ± 0.1	3.6 ± 0.3	1.9 ± 0.4	4.6 ± 0.8	1.7 ± 0.4
Total power (ms^2^)	5.5 ± 1.6	2.5 ± 0.2	11.7 ± 3.1	2.8 ± 1	13.9 ± 3.5	1.8 ± 0.9
HF (ms^2^)	2 ± 0.4	2.2 ± 0.2	7.7 ± 1.6	1.2 ± 0.5	0.8 ± 0.2	0.3 ± 0.1
LF (ms^2^)	0.4 ± 0.2	0.2 ± 0	1.2 ± 0.6	0.1 ± 0.1	0.1 ± 0	0.1 ± 0
VLF (ms^2^)	2.5 ± 0.9	0.1 ± 0	2.1 ± 0.9	1.1 ± 0.6	9.4 ± 2.4	1 ± 0.6
HF norm ()	42.1 ± 3.3	85.4 ± 0.6	69.5 ± 3.1	57.3 ± 9.8	17.7 ± 6.2	34 ± 8.6
LF norm ()	5.6 ± 0.6	7.7 ± 0.6	7.2 ± 0.9	6.4 ± 2	1.7 ± 0.6	8 ± 2.4
VLF norm ()	39.9 ± 3	5.2 ± 0.3	17 ± 2.3	27.1 ± 7.8	57.4 ± 5.6	43.9 ± 6.8
HF norm by basal ()		61.3 ± 5.8	148.5 ± 17.3		85.1 ± 28.5	14.5 ± 3.2
LF norm by basal ()		5.3 ± 0.7	15.8 ± 4.1		7.5 ± 2.6	3.6 ± 1.2
VLF norm by basal ()		3.7 ± 0.4	36.2 ± 6.4		971 ± 407	26.7 ± 7.9

*BSL, basal; ABK, autonomic blockade; AVNN, average NN interval; SDNN, SD of NN interval; HF, high frequency; LF, low frequency; VLF, very low frequency.*

To further support these conclusions, the short- and long-range HRVs were quantified by calculating sample entropy (SE), a measure of the irregularity of a signal (Richman and Moorman, 2000; Costa et al., 2008). Lower SE represents a regular signal with low variability and vice versa. To obtain multi-scale sample entropy (MSE), SE was calculated on multiple time scales, where the heartbeat interval time series for scale *s* was derived from the original time series by averaging *s* consecutive samples. Thus, the SE of an order of one is the difference between conjunctive beats. Higher scales (10 and above) represent longer-range temporal phenomena and shorter scales (1–10) represent short-range temporal phenomena (Richman and Moorman, 2000; Costa et al., 2008). In Figures 2A,B, the SE is plotted as a function of scale. Under basal conditions, both short- and long-range MSE were similar between dogs and mice. Under ABK conditions, the plots support the previous conclusions, namely, that the source of short- and long-range HRVs differs between mammals, i.e., in dogs, the extrinsic and intrinsic pacemaker mechanisms are the main contributor to long-range HRV, while the ANS is the main contributor to short-range HRV. In mice, the interaction between the ANS and extrinsic and intrinsic pacemaker mechanisms affects both short- and long-range HRVs.

Finally, we tested whether knowledge of the relative contributions of ANS and pacemaker mechanisms to HRV on different time scales can support the design of a filter that removes the ANS contribution from basal data, to obtain a signal similar to that obtained under ABK, but without drug intervention. This signal has been defined before as the extrinsic and intrinsic pacemaker mechanism signature (Rosenberg et al., 2020) and was shown to be obtainable in dogs without any interventions (Figure 2A). Figure 2B shows that by applying an FIR band-stop filter to isolate the ANS contribution to mouse recordings, the MSE curve and the power in each frequency band of filtered heartbeat interval data were similar to those obtained upon ABK achieved by drug intervention. Thus, in awake dogs and mice, the ANS and pacemaker mechanism signatures can be obtained without any drug intervention or *ex vivo* experiments (opening the Schrödinger box).

### Comparing the ANS Signature in Awake vs. Anesthetized Mice

We next tested whether ANS and pacemaker mechanism signatures can be obtained under anesthesia, without any drug intervention, or in *ex vivo* experiments. More specifically, we explored the role of extrinsic and intrinsic pacemaker mechanisms and ANS signaling on short- and long-range HRVs of anesthetized mice (*n* = 8, aged 2–4 months) (Yaniv et al., 2016), measured under basal and ABK conditions and compared them to the profiles of awake mice (*n* = 5, aged 2–4 months) (Moen et al., 2019). As illustrated in the Poincaré plot in Figure 1A and calculated by SDNN (Table 1), there was no substantial change in heartbeat interval scattering between anesthetized and awake mice. Similarly, the SDNN in the heartbeat interval between basal and ABK was not significantly changed in anesthetized mice.

In anesthetized mice, even after ABK, both LF and HF peaks were apparent (Figure 1B). Figure 2C shows that in basal-state anesthetized mice, the power was almost equally distributed between the VLF and HF bands, with less than 6% in the LF band. ABK led to a relative decrease in the VLF, shifting the majority of the power to the HF band. Thus, in anesthetized mice, the extrinsic and intrinsic pacemaker mechanisms mainly affect the short-range time scale and impact the long-range time scales to a lesser extent. In contrast, the ANS affected both time ranges in anesthetized mice. Under basal conditions, anesthetized mice had a high entropy in the short-range as compared to the long-range correlations, in sharp contrast to the trend seen in awake mice (Figure 2B), supporting the previous conclusions concerning the source of short- and long-range HRVs.

We next assessed whether a filter that removes the ANS contribution to HRV, can also be applied to data collected from anesthetized mice. Figure 2C shows that in anesthetized mice, similar to awake mice, application of an FIR band-stop filter to ANS contribution (with different parameters than the awake mice), yielded an MSE curve and power in each frequency band of filtered heartbeat interval data that were similar to those obtained under drug-induced ABK.

### Filtering the ANS Signature From Heartbeat Intervals of Anesthetized Animals

Next, we sought to determine whether pacemaker mechanisms and ANS signatures can be extracted from ECG recordings collected from anesthetized rabbits (*n* = 6, aged 2–4 months) without any drug intervention or *ex vivo* experiments (Yaniv et al., 2016) under basal and ABK conditions and compared them to the above data of anesthetized mice (*n* = 8, aged 2–4 months) (Yaniv et al., 2016). Under basal conditions, the Poincaré plot (Figure 1A) and SDNN (Table 1) showed lower heartbeat interval scattering in anesthetized rabbits as compared to mice. However, as was seen in mice, the SDNN in the heartbeat interval was not significantly different between ABK and basal states of the tested rabbits.

Autonomic blockade abolished the peaks in LF and HF in anesthetized rabbits (Figure 1B). Figure 2D shows that in anesthetized rabbits, under the basal state, the majority of the PSD was found in the VLF and HF bands. ABK abolished the relative power in the HF band and shifted the majority of it in the VLF band, with some remaining in the LF band. Thus, in rabbits under anesthesia, the extrinsic and intrinsic pacemaker mechanisms mostly affect the long-range time scales and impact the short-range time scales together with ANS to a lesser extent. Under basal conditions, there was high entropy in the short-range rather than in the long-range time scale in anesthetized rabbits (Figure 2D), similar to what was observed in anesthetized mice. The plots generated from data collected under ABK conditions, support the previous conclusions regarding the source of short- and long-range HRVs. Figure 2D shows that by applying an FIR band-stop filter to ANS contribution, the MSE curve and the power in each frequency band of filtered heartbeat interval data of rabbits are similar to those obtained with data collected under drug-induced ABK.

### Distinguishing the Contribution of Extrinsic vs. Intrinsic Pacemaker Mechanisms to the Heartbeat Interval

Finally, to estimate the relative contribution of the extrinsic vs. intrinsic pacemaker mechanisms to the heartbeat interval of each mammal, we compared the ABK data to published results on isolated tissue (Shemla et al., 2021) or isolated heart (Frey et al., 1996). As shown in Figure 2E, in the isolated sinoatrial node (SAN) from mice (including only the intrinsic pacemaker mechanisms), the majority of PSD was in the LF regime, and the remaining power was in the VLF (Shemla et al., 2021). Thus, for mice, extrinsic mechanisms shift the power from LF to VLF under ABK conditions. In isolated SAN from rabbit (Shemla et al., 2021) (as shown in Figure 2F) or isolated heart (Frey et al., 1996), the majority of PSD was in the VLF regime. Thus, for rabbits, the extrinsic contribution is minor compared to intrinsic pacemaker mechanisms.

## Discussion

By using ECG data collected before and after ABK, we showed that in dogs, rabbits, and either awake or anesthetized mice, the ANS and extrinsic and intrinsic pacemaker mechanism signatures can be obtained from heartbeat intervals, without the need for any drug intervention or *ex vivo* experiments (opening the Schrödinger box). In addition, the relative contribution of each mechanism to short- and long-range HRVs is mammal-specific, and, in mice, depends on the experimental conditions (awake vs. anesthetized).

The novel method for extraction of and the distinction between the contribution of ANS and extrinsic and intrinsic pacemaker mechanisms on HRV from heartbeat interval of dogs, rabbits, and either awake or anesthetized mice, precludes the need to isolate the SAN tissue or for drug-induced ABK and allows us to better understand interactions between ANS and pacemaker mechanics. Although the filter needs to be adapted to each specific mammal, the present analysis involved the main animal models used today in physiological research. Moreover, the method opens a new clinical tool to understand the mechanisms underlying changes in HRV, which can be of high value in developing more specific treatments. We compared the performance of the designed FIR filter to a simple low-pass filter that aims to preserve the VLF band only. Supplementary Figure 2 and Supplementary Table 1 show that for dogs, awake mice, anesthetized mice, and anesthetized rabbits, the FIR filter setting is superior to the VLF filter because the filtered data values were closer to the ABK data.

Short- and long-range HRVs in mice were shown to be dependent on the experimental conditions (awake vs. anesthetized). While in awake mice, both short- and long-range time scales were affected by both ANS and extrinsic and intrinsic pacemaker mechanisms, and, in anesthetized mice, there was a clear separation between the contribution of these systems. The extrinsic and intrinsic pacemaker mostly affected the short-range time scales, while the ANS affected both time scales. Thus, the anesthesia shifted the extrinsic and intrinsic pacemaker activity toward the short-range time scale, affecting the ANS system as well. Note that the anesthesia reduced the power in all bands. Thus, the power in HF did not increase; only its relative contributions increased.

The observed contributions of the ANS vs. extrinsic and intrinsic pacemaker mechanisms to short- and long-range HRVs were mammal-specific. First, the mice and dogs were compared that were measured under awake conditions. While in dogs, the long-range time scales were mostly affected by the extrinsic and intrinsic pacemaker mechanisms, in awake mice, both short- and long-range time scales were shaped by both ANS and pacemaker mechanisms. One of the reasons for such behavior in mice may be related to the balance between sympathetic and parasympathetic activity and the magnitude of activity of each in different mammals. Mice have a higher sympathetic activity that leads to a higher beating rate as compared to dogs. Note that mice under the provided housing conditions (20°C, well below the thermoneutrality temperature) showed even higher sympathetic activity, which impacts LF more than HF (Axsom et al., 2020). When comparing mice and rabbits under anesthesia, the extrinsic and intrinsic pacemaker mostly affected the long-range time scales in rabbits but mostly affected the short-range time scales together with the ANS in mice. Thus, the anesthesia affects both ANS and pacemaker mechanisms and the interaction between them on the short-time range scale.

The contribution of extrinsic pacemaker mechanisms to short- and long-range HRVs is also mammal-specific. While their contribution was negligible in rabbits, in mice, extrinsic mechanisms shifted the internal pacemaker mechanisms to contribute to both short- and long-time scales. Comparing other mammals under ABK to isolated SAN data will enable evaluation of the influence of the extrinsic pacemaker on HRV and may enable the design of a universal filter to isolate their influence on HRV.

**FIGURE 3:**
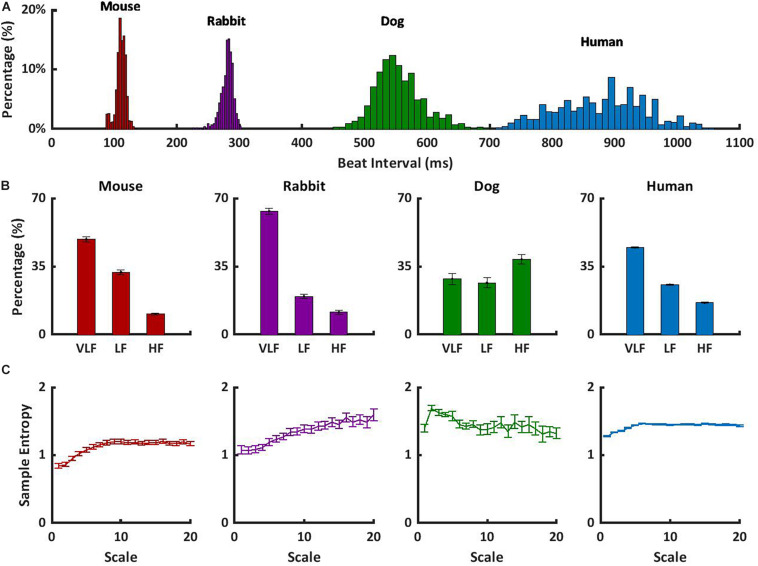
Short- and long-range HRVs in basal HRV in awake mammals. **(A)** Heartbeat interval histogram for mice, rabbits, dogs, and humans. **(B)** Normalized power in the VLF, LF, and HF bands, and **(C)** average multiscale entropy. Frequency and multiscale entropy dog results are from Rosenberg et al. (2020).

To understand how to translate the animal results to human data, we observed the short- and long-range HRVs of awake mammals, including humans, under basal conditions. In addition to the data presented here, we explored the short- and long-range HRVs in humans (*n* = 18) (Goldberger et al., 2000). Figure 3A shows that as the heartbeat interval increases, the variability increases. These results are in accordance with the reverse nonlinear relationship between average beating rate and HRV (Rocchetti et al., 2000; Zaza and Lombardi, 2001). In humans, the majority of the PSD was contained in the VLF and LF bands, with a ratio of 2:1 between LF and HF, as previously documented (Nunan et al., 2010; Figure 3B). Similar trends were noted here in rabbits and mice. The differences in the content of the HF-band PSDs between humans and dogs may be related to different rates of respiratory sinus arrhythmia, which is the main mechanism affecting the HF band. Because ABK leads to a decrease in heartbeat interval in dogs similar to that observed in humans, and because the pacemaker signature does not affect the long-range HRV (Figure 3C), the dog model seems to ideally represent the human pacemaker function, the rabbit seems like an ideal model for ANS function study, and the mouse seems like a model when interactions between both the ABK and pacemaker mechanisms are observed.

### Limitations

The “general filtering” method of ANS contribution needs to be adapted for each specific mammal. For a new mammal model, short- and long-range HRVs analysis must be performed on both basal and ABK data to calibrate the filter parameters. Future testing on transgenic animals must be performed to determine whether the filter needs further adaptation per species.

The filter performance was tested using MSE analysis. Note that the MSE method has two main limitations that have been previously identified (Valencia et al., 2009): (i) filtering of some information in the VLF and (ii) simplification of the model (assuming the physiological model can be described by coarse-graining model).

In any biological system, noise and artifacts can affect the PSD. This source of noise mainly affects the VLF power and thus cannot be ruled out.

While data collected under different experimental conditions were compared here, the filter approach was tested on the same mammal under the same conditions and therefore provides valuable information.

## Conclusion

While the ANS contributes to short-time range HRV in dogs, in rabbits, and mainly in mice, its interactions with extrinsic and intrinsic pacemaker mechanisms also impact short-time range HRV. Thus, distinct short- and long-range HRVs are seen in different mammals, each shaped by different sets of mechanisms and experimental conditions. Therefore, animal models for the exploration of ANS or pacemaker mechanisms must be carefully selected. Note, that factors other than the ANS and internal pacemaker mechanisms, e.g., circulating hormones (e.g., endocrine), contribute to HRV. By comparing heartbeat interval under ABK to isolate SAN data, we found that the extrinsic pacemaker system affects the short- and long-range HRVs differently.

## Data Availability Statement

Publicly available datasets were analyzed in this study. This data can be found here: Published ECG data collected from dogs (Billman et al., 2015), rabbits (Yaniv et al., 2014), mice (Yaniv et al., 2016; Moen et al., 2019), and humans (Goldberger et al., 2000) were used.

## Ethics Statement

The animal study was reviewed and approved by Published ECG data collected from dogs (Billman et al., 2015), rabbits (Yaniv et al., 2014), mice (Yaniv et al., 2016; Moen et al., 2019), and humans (Goldberger et al., 2000) were used. The protocols and experimental procedures were approved by the original research committees overseeing the studies described in the respective publications.

## Author Contributions

IW-B and YY conceived and designed the research. IW-B analyzed the databases with supervision of AR. IW-B and MD designed the filters. AA designed the analysis interface and scripts for data arrangement. YY drafted the manuscript. IW-B, MD, AR, and AA edited and revised the manuscript. YY, IW-B, MD, AR, and AA approved the final version. All authors contributed to the article and approved the submitted version.

## Conflict of Interest

The authors declare that the research was conducted in the absence of any commercial or financial relationships that could be construed as a potential conflict of interest.

## Abbreviations

ABK: autonomic nervous system blockade
ANS: autonomic nervous system
FIR: finite impulse response
HF: high frequency
HRV: heart rate variability
LF: low frequency
MSE: multi-scale sample entropy
PSD: power spectral density
SAN: sinoatrial node
SE: sample entropy
VLF: very low frequency.
