# Supplementary material for: Opening the Schrödinger Box: Short- and Long-Range Mammalian Heart Rate Variability
Source: Front Physiol. 2021 Jun 30;12:665709. doi: 10.3389/fphys.2021.665709 (PMC8278020; doi:10.3389/fphys.2021.665709)
Supplement: Supplementary file 1 [file Data_Sheet_1.DOCX]

**Brief Research Report**

**Opening the Schrödinger box: Short- and long-range mammalian heart rate variability**

**Supplement**

Running title: Mammalian heart rate variability

Ido Weiser-Bitoun^1^, Moran Davoodi^1^, Aviv A. Rosenberg^2^, Alexandra Alexandrovich^1^

and Yael Yaniv^1^

^1^Biomedical Engineering Faculty, ^2^Computer Science Faculty Technion-IIT, Haifa, Israel 32000

*Correspondence to:

Yael Yaniv, PhD
Laboratory of Bioenergetic and Bioelectric Systems,
Biomedical Engineering Faculty, Technion – Israel Institute of Technology,
Haifa, Israel

Email: [yaely@bm.technion.ac.il](mailto:yaely@bm.technion.ac.il)
Phone: 972-4-8294124
Fax: 972-4-8294599

**Keywords:** Animals, Autonomic Blockade, Cardiac disease, ECG, Sinoatrial node

**Table S1:** HRV measures for different mammals. BSL-basal, ABK- Autonomic blockade**,** AVNN-Average NN interval, SDNN-Standard deviation of NN interval, HF-high frequency, LF-low frequency, VLF-very low frequency.

|  | Dogs (n=14) | | | Awake Mice (n=5) | | |
| --- | --- | --- | --- | --- | --- | --- |
|  | **ABK** | **FIR Filter** | **VLF Filter** | **ABK** | **FIR Filter** | **VLF Filter** |
| AVNN (ms) | 460.3±11.3 | 486.6±13.4 | 498.2±17.6 | 118.6±1 | 110.5±1.4 | 110.3±1.3 |
| SDNN (ms) | 21.6±2.1 | 31.5±2.7 | 38.2±3.4 | 4.7±0.2 | 9.3±0.4 | 8.4±0.3 |
| Total Power (ms^2^) | 247.4±47.3 | 880.5±233.4 | 1234.1±244.7 | 23.1±2.6 | 79.7±7.1 | 64.9±6.1 |
| HF (ms^2^) | 12.5±6.1 | 9.6±3.3 | 0.3±0.1 | 2.3±0.2 | 10.3±0.8 | 0±0 |
| LF (ms^2^) | 18.8±3.9 | 12.5±1.9 | 45.7±8.4 | 3.6±0.5 | 3.6±0.3 | 0.8±0.2 |
| VLF (ms^2^) | 139.3±30 | 779.9±215.4 | 1127.2±237 | 14.4±1.8 | 55.3±5.6 | 54.7±5.3 |
| HF norm () | 4.3±1.6 | 2.4±0.9 | 0±0 | 16.6±1 | 15.7±0.9 | 0±0 |
| LF norm () | 9.8±1.3 | 2.7±0.5 | 8.2±2 | 12.6±0.9 | 6.2±0.4 | 2.2±0.5 |
| VLF norm () | 59.8±3.4 | 85.6±1.3 | 86.4±1.8 | 58.6±1.4 | 66.2±1.3 | 84.2±1 |
| HF norm by basal () | 0.4±0.2 | 0.4±0.2 | 0±0 | 2.1±0.2 | 9.3±0.7 | 0±0 |
| LF norm by basal () | 0.5±0.1 | 0.5±0.1 | 2.3±0.6 | 3.7±0.6 | 3.5±0.3 | 0.8±0.2 |
| VLF norm by basal () | 4.3±1.3 | 29.1±4.9 | 35.6±4.5 | 12.6±1.3 | 49.9±4.5 | 49.1±4.2 |
|  | **Anesthetized Mice (n=8)** | | | **Anesthetized Rabbits (n=7)** | | |
|  | **ABK** | **FIR Filter** | **VLF Filter** | **ABK** | **FIR Filter** | **VLF Filter** |
| AVNN (ms) | 131.3±0.4 | 125.5±0.8 | 125.5±0.8 | 204.6±3.7 | 192.8±5.8 | 188.4±5.7 |
| SDNN (ms) | 1.7±0.1 | 3.6±0.3 | 1.8±0.2 | 4.6±0.8 | 1.7±0.4 | 1.3±0.4 |
| Total Power (ms^2^) | 2.5±0.2 | 11.7±3.1 | 3±1 | 13.9±3.5 | 1.8±0.9 | 1.5±0.8 |
| HF (ms^2^) | 2.2±0.2 | 7.7±1.6 | 0±0 | 0.8±0.2 | 0.3±0.1 | 0±0 |
| LF (ms^2^) | 0.2±0 | 1.2±0.6 | 0±0 | 0.1±0 | 0.1±0 | 0±0 |
| VLF (ms^2^) | 0.1±0 | 2.1±0.9 | 2.4±0.9 | 9.4±2.4 | 1±0.6 | 1±0.6 |
| HF norm () | 85.4±0.6 | 69.5±3.1 | 0±0 | 17.7±6.2 | 34±8.6 | 0.1±0.1 |
| LF norm () | 7.7±0.6 | 7.2±0.9 | 0±0 | 1.7±0.6 | 8±2.4 | 11.2±6.5 |
| VLF norm () | 5.2±0.3 | 17±2.3 | 76.6±3.3 | 57.4±5.6 | 43.9±6.8 | 67.9±4 |
| HF norm by basal () | 61.3±5.8 | 148.5±17.3 | 0±0 | 85.1±28.5 | 14.5±3.2 | 0±0 |
| LF norm by basal () | 5.3±0.7 | 15.8±4.1 | 0±0 | 7.5±2.6 | 3.6±1.2 | 1.5±0.7 |
| VLF norm by basal () | 3.7±0.4 | 36.2±6.4 | 41.4±6.5 | 971±407 | 26.7±7.9 | 27±7.9 |

**
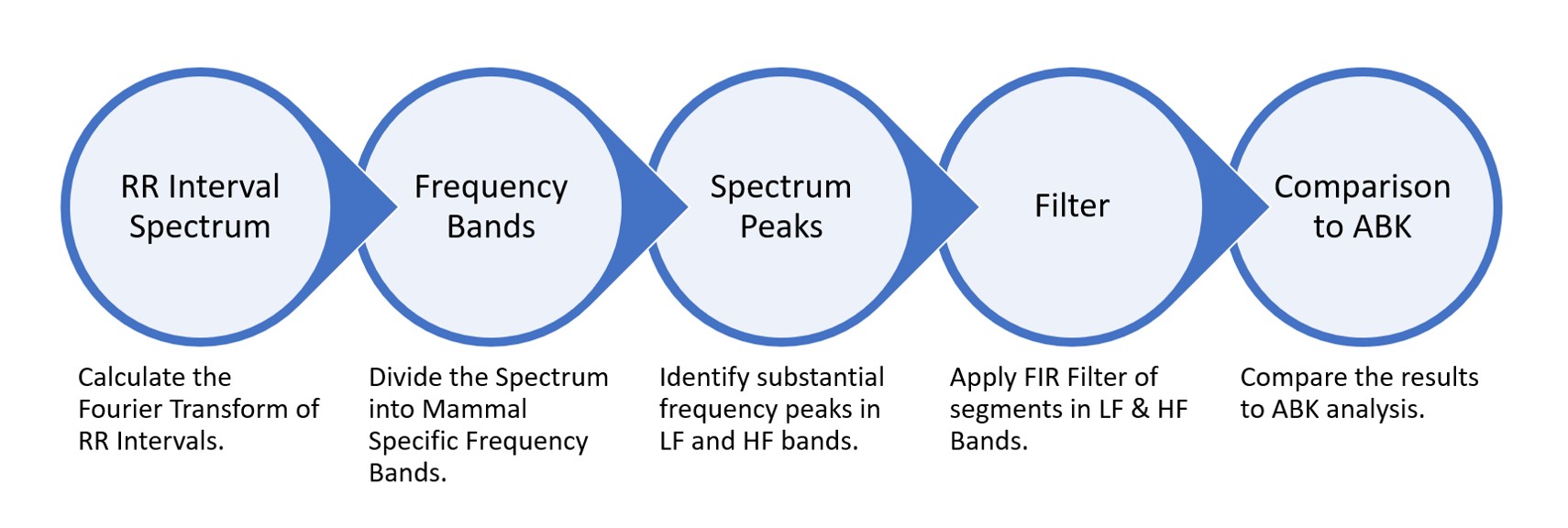
**

**Figure S1.** Generic method to isolate the ANS contribution to mammalian ECG data.


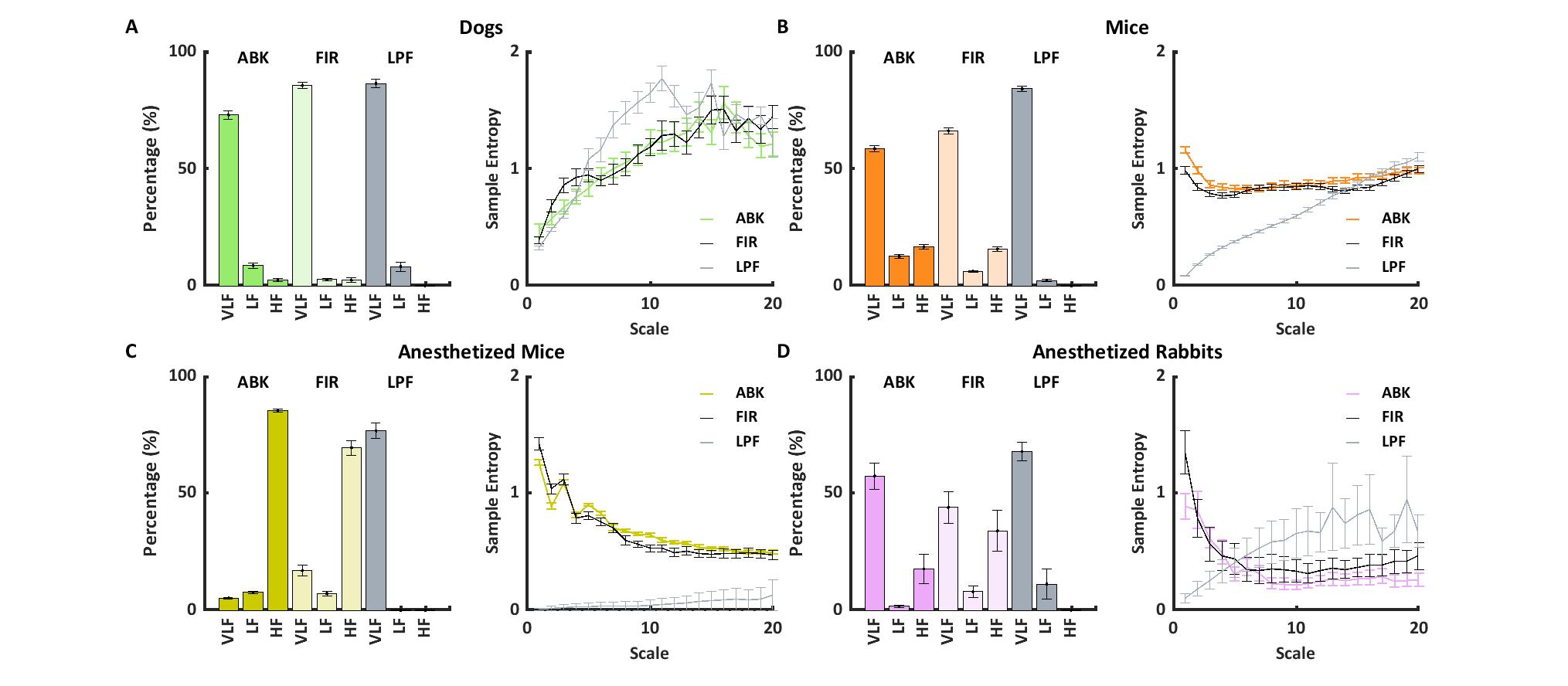


**Figure S2. Short- and long-range HRVs.** Normalized power in the very low frequency (VLF), low frequency (LF) and high frequency (HF) bands and average multiscale entropy of autonomic nervous blockade **(**ABK), FIR filter and lowpass filter (LPF) in (A) dogs (n=14, aged 1-4 years), (B) awake mice (n=5, aged 2-4 months), (C) anesthetized mice (n=6, aged 2-4 months) and (D) anesthetized rabbits (n=8, aged 2-4 months). Dog ABK results are from (Rosenberg et al., 2020).
